# Intestinal overgrowth of *Clostridium perfringens* and reduced *cadA* gene copy number characterize fatal diarrhea in captive corn snakes (*Pantherophis guttatus*)

**DOI:** 10.3389/fvets.2026.1812888

**Published:** 2026-05-25

**Authors:** You-Chen Hung, Wen-Yuan Yang

**Affiliations:** 1 Department of Veterinary Medicine, School of Veterinary Medicine, National Taiwan University, Taipei, Taiwan; 2 Zoonoses Research Center and School of Veterinary Medicine, National Taiwan University, Taipei, Taiwan

**Keywords:** *cadA* gene, *Clostridium perfringens*, corn snakes, fatal diarrhea, intestinal overgrowth, *Pantherophis guttatus*, reptile enteric disease, virulence genes

## Abstract

**Introduction:**

Fatal diarrhea has recently emerged as a significant health problem in captive-bred corn snakes (*Pantherophis guttatus*), particularly among juveniles under 6 months of age. Affected snakes typically present with watery diarrhea, anorexia, dehydration, and high mortality.

**Methods:**

To clarify the etiological basis of this syndrome, we investigated bacterial pathogens associated with fatal diarrhea and characterized virulence gene profiles of predominant isolates. Rectal swabs were collected from healthy and diarrheic snakes, and pathogen detection and bacterial loads were quantified.

**Results:**

*Clostridium perfringens* demonstrated a significantly higher detection rate and rectal bacterial load in diarrheic snakes compared with healthy individuals (*p* = 0.0007; *p* < 0.0001), whereas *Salmonella* spp. and *Escherichia coli* were not associated with disease status. Toxinotyping of 24 *C. perfringens* isolates identified toxinotypes A, B, D, and G without a disease-specific distribution, suggesting that toxinotype alone is insufficient to explain outbreak occurrence. Quantitative analysis of five virulence-associated genes, including cell-wall-anchored extracellular nuclease (*cadA*), collagen adhesion protein (*cna*), sialidase (*nanI*), necrotic enteritis B-like toxin (*netB*), and toxin *C. perfringens* large cytotoxin (*tpeL*) genes, revealed a significantly lower *cadA* gene copy number in isolates from diarrheic snakes (*p* = 0.0343), while no significant differences were observed for the other genes.

**Discussion:**

Fatal diarrhea in captive corn snakes is associated with increased intestinal colonization by *C. perfringens*, rather than with toxinotype variation. The reduced *cadA* gene copy number observed in isolates from diarrheic snakes suggests a potential role for biofilm-related mechanisms in disease expression, although functional studies are required for confirmation. These findings provide field-based evidence supporting the involvement of *C. perfringens* overgrowth in reptile enteric disease and may inform future disease monitoring and management strategies in intensive breeding systems.

## Introduction

Captive breeding of corn snakes (*Pantherophis guttatus*) has grown rapidly in Asia alongside the global growth of the reptile pet trade. Native to the United States and first domesticated as pets in the 1960s, corn snakes are now ranked as the fourth most kept reptile worldwide (1). The increasing popularity of reptiles as companion animals has led to growing concern regarding their associated health issues. Reptiles are recognized reservoirs of diverse enteric bacteria, including zoonotic and opportunistic pathogens, which may contribute to gastrointestinal disease under certain conditions (2). Recent reports from different regions further highlight the clinical significance of bacterial infections in captive reptiles. In China, an outbreak of pneumonia and enteritis in a snake farm with more than 3,000 snakes was associated with *Salmonella* and other bacteria co-infections (3). In Brazil, eight cases of fibrinonecrotic or granulomatous enterocolitis was reported in night captive snakes, demonstrating the link of bacterial infection with enteric lesions (4). In addition, a case from Canada described 7 days diarrhea associated with enterotoxemic *Clostridium perfringens* infection in a captive red-footed tortoise (5). These findings indicate that gastrointestinal disorders, including diarrhea, are not uncommon in captive reptile populations and may arise from bacterial pathogens under certain mechanisms.

Approximately 75% of diseases in snakes are attributed to bacterial infections, some of which arise from opportunistic organisms that are part of the normal intestinal microbiota (3). Several studies have demonstrated that reptiles commonly harbor enteric bacteria such as *Salmonella* spp., *Escherichia coli*, and *C. perfringens*, indicating that the reptile gastrointestinal tract can support colonization by potentially pathogenic organisms (2, 6, 7). Clinical cases further suggest that these bacteria may contribute to disease under specific conditions. For example, *Salmonella* infection has been associated with necrotizing gastritis in snakes (4). *E. coli*, a common intestinal pathogen in animals, is readily isolated from corn snake gastrointestinal contents, and both pathogenic and non-pathogenic strains have been documented (6). Additionally, *C. perfringens* enterotoxin (CPE) toxin-producing strain has been implicated in enteric disease in reptiles, including a case of diarrhea in a captive red-footed tortoise (*Chelonoidis carbonaria*) (5). To date, most studies on reptile enteric disease have primarily focused on *Salmonella* infections, with relatively limited attention given to the potential role of *C. perfringens*. Notably, in the reported case of *C. perfringens*-associated diarrhea, other common enteric pathogens such as *Salmonella* spp. and *C. difficile* were not detected, suggesting that *C. perfringens* may act as a primary etiological agent under certain conditions. These findings indicate that *C. perfringens* should be considered in the differential diagnosis of diarrheal disease in reptiles.

In Taiwan, breeders have recently reported recurrent outbreaks of fatal diarrhea in captive corn snakes. Diarrhea is most observed in snakes aged 6–8 months with highest mortality occurring in snakes younger than 6 months of age. Affected snakes typically develop watery diarrhea, anorexia, dehydration, growth retardation, and progressive emaciation. Mortality often occurs within 15 days after disease onset, whereas surviving snakes frequently develop chronic diarrhea. Although these observations indicate the presence of both acute and chronic disease forms, detailed epidemiological and microbiological information on this syndrome is currently limited, and its underlying etiology warrants further investigation.

Bacterial virulence genes play critical roles in host colonization, persistence, and disease progression. These factors include proteins involved in adhesion, tissue invasion, nutrient acquisition, and modulation of host responses. In *C. perfringens*, for example, collagen adhesion proteins facilitate attachment to host tissues (8), while sialidases degrade host glycans and provide nutrients that support bacterial growth (9, 10). In addition, genes such as cell-wall-anchored extracellular nuclease (*cadA*) have been associated with biofilm formation and may contribute to bacterial persistence in the intestinal environment (11, 12). However, the presence and distribution of these virulence-associated traits in reptile isolates, and their relationship with disease status, remain poorly characterized.

Although several bacterial species have been detected in reptiles, most previous studies have primarily focused on pathogen prevalence rather than quantitative assessment or detailed characterization of virulence features (2, 6, 13, 14). As a result, the relationships among bacterial loads, virulence gene profiles, and clinical outcomes remain unclear. Therefore, the present study aimed to investigate the bacterial agents associated with fatal diarrhea in captive corn snakes through a multi-facility field investigation. Specifically, we compared intestinal bacterial loads between healthy and diarrheic snakes and further characterized bacterial genotypes and selected virulent genes of the isolates. This integrated approach was designed to improve understanding of pathogen involvement in disease development and to identify potential factors associated with clinical outcomes.

## Methods

### Sample collection

All snakes included in this study were of similar age (approximately 8 months) and body weight (15–25 g). Snakes in the healthy group had no history of diarrhea throughout the entire rearing period prior to sampling. Snakes in the diarrheic group were defined as individuals of the same age that had exhibited continuous diarrhea for more than 7 consecutive days at the time of sample collection. Corn snakes were individually housed in rack systems across all farms. Within each farm, snakes from both healthy and diarrheic groups were maintained under comparable housing conditions, including similar enclosure sizes and management practices.

**Figure 1:**
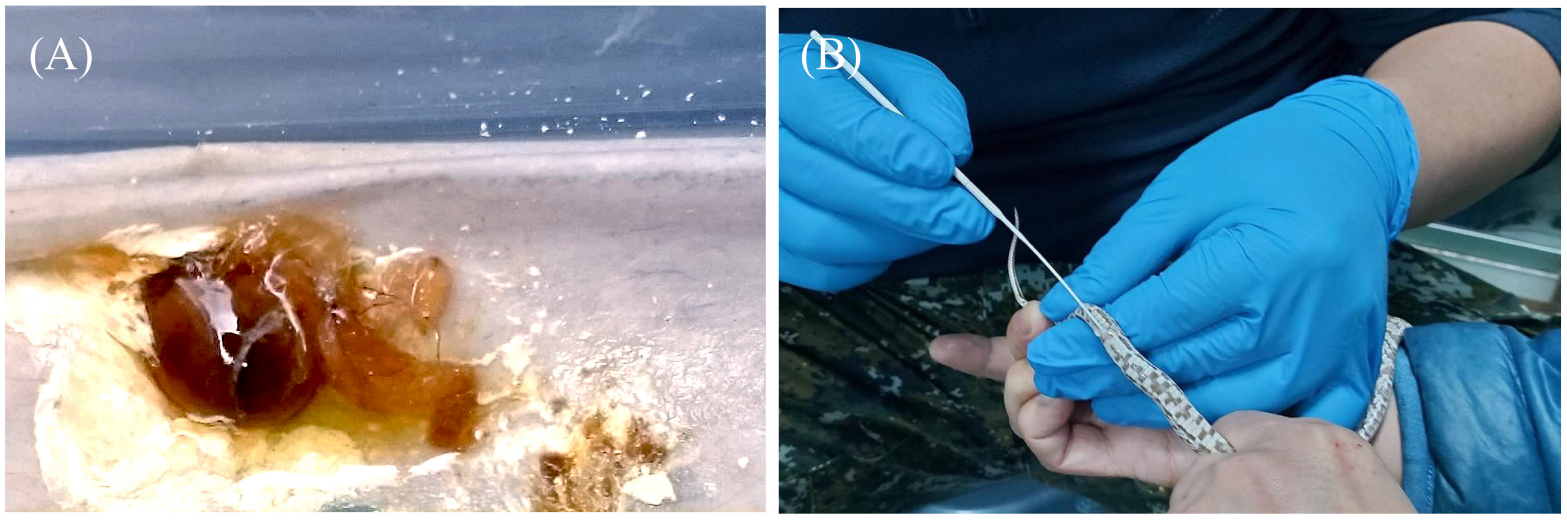
*Clinical presentation of diarrhea and rectal swab collection in captive corn snakes* (*Pantherophis guttatus*). **(A)** Watery feces with visible mucus and sloughed intestinal material. **(B)** Rectal swab collection procedure: the swab tip was moistened with sterile phosphate-buffered saline, inserted approximately 1 cm into the rectum, rotated to ensure mucosal contact, and withdrawn to collect mucosal material.

In the first phase, rectal samples were collected for bacterial isolation using sterile nylon-flocked swabs inserted through the cloaca into the rectum. The objective of this phase was to compare the abundance of bacterial species between healthy and diarrheic corn snakes and to identify bacteria associated with diarrhea. To exclude transient diarrhea related to short-term husbandry or environmental changes, diarrheic cases were defined as snakes that showed watery feces for more than 7 consecutive days, with recurrent episodes accompanied by visible intestinal mucus or sloughed mucosa, anorexia, weakness, and dehydration. Snakes with any history of medical treatment or antibiotic use were excluded. Healthy snakes were defined as animals with no history of diarrhea, normal hydration, good appetite, and well-formed feces. Four farms with ongoing diarrhea outbreaks were included in this phase. From each farm, rectal swabs were collected from 10 diarrheic and 10 healthy snakes, which yielded a total of 40 samples per health status. Sampling was conducted by collecting samples from healthy snakes first, followed by diarrheic snakes on the same farm. Before sampling, swab tips were moistened with sterile phosphate-buffered saline (PBS). Each swab was inserted approximately 1 cm into the rectum and gently rotated to collect mucosal material (Figure 1). Swab tips were placed into sterile tubes containing 3 mL of PBS and fully submerged. All samples were transported to the laboratory on the same day at ambient temperature for bacterial culture and comparative analysis.

In the second phase, eight additional farms that met the same case definition were enrolled. Sampling followed the same protocol as in the first phase, with 10 healthy and 10 diarrheic snakes sampled per farm. One representative *C. perfringens* isolate per health status (diarrheic or healthy) per farm was randomly selected from each farm for further analysis. Together with isolates obtained during the first phase, a total of 24 *C. perfringens* isolates were included, comprising 12 isolates from healthy snakes and 12 from diarrheic snakes, for virulence gene profiling. All 12 farms operated under comparable intensive husbandry conditions. Snakes were housed individually in plastic enclosures within commercial rack systems lined with aspen shavings. Each enclosure contained a water dish, and heat was supplied by thermostatically controlled heat tape. Sick snakes were housed separately from healthy individuals on each farm. Farm populations ranged from approximately 1,000 to 1,500 corn snakes.

### Pathogen screening and bacterial enumeration

Rectal swab samples preserved in 3 mL PBS were vortexed for 30 s at room temperature. An aliquot of 100 μL from each homogenized suspension was plated onto selective agar media and incubated at 37°C for 20 h to allow colony enumeration. *Salmonella* spp. was isolated in accordance with ISO 6579-1 guidelines using xylose lysine deoxycholate agar (Himedia, Mumbai, India) and brilliant green phenol red lactose sucrose agar (Himedia, Mumbai, India). *E. coli* was isolated on eosin methylene blue agar (Himedia, Mumbai, India). *C. perfringens* was isolated using tryptose sulfite cycloserine agar (Himedia, Mumbai, India) and confirmed on sheep blood agar under anaerobic conditions. Reference strains of *Salmonella* Enteritidis, *E. coli* ATCC 25922, and *C. perfringens* ATCC 27324 were included as positive controls to confirm culture performance and colony morphology. Detection rates and bacterial counts were recorded for each target species and used for subsequent comparative analyses.

### Molecular characterization

Based on the results of bacterial screening and comparative analysis between diarrheic and healthy snakes, *C. perfringens* was selected for further molecular characterization of virulence-associated genes and toxinotypes. *C. perfringens* isolates obtained from phases one and two were enriched anaerobically in fluid thioglycollate medium (Himedia, Mumbai, India) at 37°C for 18 h. Genomic DNA was extracted from 1 mL of bacterial culture using the DNeasy Blood and Tissue Kit (Qiagen, NRW, Germany) according to the manufacturer's protocol. Species identification was confirmed by PCR amplification of a *C. perfringens*-specific *16S rRNA* gene fragment. Toxinotyping was performed by PCR detection of toxin genes that define established *C. perfringens* toxinotypes (15). Reference strains ATCC 27324, ATCC 3626, and the field isolate JP18 were included as positive controls. Primer sequences used for species confirmation and toxinotyping are listed in Table S1.

### Quantitative analysis of virulence genes in isolates

Five virulence genes, including cell-wall-anchored extracellular nuclease (*cadA*), collagen adhesion protein (*can*), sialidase (*nanI*), necrotic enteritis B-like toxin (*netB*), and toxin *C. perfringens* large cytotoxin (*tpeL*), associated with bacterial adhesion, intestinal persistence, and toxin activity were selected for quantitative comparison among *C. perfringens* isolates. The necrotic enteritis field isolate JP-17, which carries all five target genes, served as the positive control. Target gene fragments were amplified by PCR, cloned into the pGEM-T vector, and transformed into *E. coli* DH5α. Transformants were selected on LB agar (Himedia, Mumbai, India) supplemented with ampicillin, X-gal, and IPTG. White colonies were chosen for plasmid extraction, and insert identity was confirmed by Sanger sequencing using the T7 promoter primer. Purified plasmid DNA was diluted in Tris buffer to 0.5 ng/μL, followed by 10-fold serial dilutions down to 50 pg/μL to generate standard curves for each gene. All curves showed coefficients of determination (*R*^2^) ≥ 0.99, no evident outliers, and amplification efficiencies between 90 and 110% (Figure S1).

Quantitative PCR (qPCR) was performed in a final volume of 10 μL using the PowerUp™ SYBR™ Green Master Mix (Thermo Fisher Scientific, MA, USA) on a QuantStudio™ 3 Real-Time PCR System (Thermo Fisher Scientific, MA, USA). DNA concentration was measured using a NanoDrop spectrophotometer (Thermo Fisher Scientific, MA, USA), and all samples were normalized to 50 ng/μL using nuclease-free water prior to qPCR analysis. A fixed volume of 2 μL of template DNA (100 ng per reaction) was used in each qPCR assay to ensure consistency and comparability among samples. The thermal cycling protocol included an initial activation step at 95°C for 2 min, followed by 45 cycles of denaturation at 95°C for 5 s and annealing/extension at 60°C for 30 s. Cycle threshold (Ct) values were recorded, and gene copy numbers were calculated using linear regression equations derived from the corresponding standard curves.

### Statistical analysis

All statistical analyses were performed using SAS version 9.4 (SAS Institute Inc., Cary, NC, USA). Differences in detection rates between healthy and diarrheic snakes were evaluated using the Chi-square test or Fisher's exact test, as appropriate based on expected cell counts. Normality of continuous variables was assessed using the Shapiro-Wilk test. Variables with a normal distribution were compared using two-sample *t* tests, whereas variables that did not meet normality assumptions were analyzed using the Wilcoxon rank-sum test. All analyses used two-tailed tests, and statistical significance was defined as *p* < 0.05.

## Results

### Bacterial detection rates in healthy and diarrheic snakes

*Salmonella* spp., *E. coli*, and *C. perfringens* were detected in healthy snakes from all farms included in the first phase, with farm-level detection rates of 100% (4/4). At the sample level, detection rates in healthy snakes were 50% (20/40) for *Salmonella* spp., 45% (18/40) for *E. coli*, and 75% (30/40) for *C. perfringens*. The same three bacterial species were also detected in diarrheic snakes from all farms, again with farm-level detection rates of 100% (4/4). Sample-level detection rates in diarrheic snakes were 30% (12/40) for *Salmonella* spp., 37.5% (15/40) for *E. coli*, and 100% (40/40) for *C. perfringens* (Table 1). Farm-level analysis showed no significant differences between healthy and diarrheic snakes in the detection of *Salmonella* spp. or *E. coli* at any farm (all *p* > 0.05). In contrast, detection of *C. perfringens* was significantly higher in diarrheic snakes than in healthy snakes at Farm 1 (*p* = 0.0031). When data from all four farms were pooled, detection rates of *Salmonella* spp. and *E. coli* did not differ significantly between health groups (*p* = 0.0679 and *p* = 0.4957, respectively). However, *C. perfringens* detection remained significantly higher in diarrheic snakes than in healthy snakes (*p* = 0.001).

**Table 1:** Bacterial detection rates of *Salmonella spp*., *E. coli* and *C. perfringens* in the rectal swabs.

Sampling farm	Phase	*Salmonella* spp.			*E. coli*			*C. perfringens*		
		Group		*p*-value	Group		*p*-value	Group		*p*-value
		Normal	Diarrhea		Normal	Diarrhea		Normal	Diarrhea	
Farm 1	*1*	40% (4/10)	20% (2/10)	0.6285	20% (2/10)	10% (1/10)	>0.9999	30% (3/10)	100% (10/10)	*0.0031*
Farm 2	*1*	60% (6/10)	40% (4/10)	0.6563	20% (2/10)	30% (3/10)	>0.9999	100% (10/10)	100% (10/10)	>0.9999
Farm 3	*1*	40% (4/10)	30% (3/10)	>0.9999	70% (7/10)	60% (6/10)	>0.9999	80% (8/10)	100% (10/10)	0.4737
Farm 4	*1*	60% (6/10)	30% (3/10)	0.3698	70% (7/10)	50% (5/10)	>0.9999	90% (9/10)	100% (10/10)	>0.9999
*Total*	*-*	50% (20/40)	30% (12/40)	0.0679	45% (18/40)	37.5% (15/40)	0.4957	75% (30/40)	100% (40/40)	*0.001*

Normal: isolates from clinically healthy corn snakes without diarrhea; Diarrhea: isolates from corn snakes that met the case definition for diarrhea; n: sample size.

Differences in detection rates between normal and diarrheic groups were analyzed using the Chi-square test. Fisher's exact test was applied when expected cell counts were insufficient. Statistical significance was set at *p* < 0.05.

### Comparison of rectal bacterial loads between healthy and diarrheic snakes

Rectal bacterial loads of *Salmonella* spp., *E. coli*, and *C. perfringens* were compared between healthy and diarrheic snakes from all farms included in the first phase. At the individual farm level, bacterial loads of *Salmonella* spp. and *E. coli* did not differ significantly between healthy and diarrheic groups. In contrast, *C. perfringens* loads were significantly higher in diarrheic snakes than in healthy snakes at all four farms (*p* = 0.0001, *p* = 0.0014, *p* = 0.0005, and *p* = 0.0007, respectively). When data from all four farms were pooled for overall analysis, no significant differences were observed between healthy and diarrheic snakes for *Salmonella* spp. (*p* = 0.0821) or *E. coli* (*p* = 0.7752). However, rectal loads of *C. perfringens* remained significantly higher in diarrheic snakes than in healthy snakes (*p* < 0.0001). Rectal bacterial loads for each farm are summarized in Table 2.

**Table 2:** Comparisons of log_10_ transformed bacterial cell counts (CFU/mL) from rectal swab samples between healthy and diarrheic corn snakes.

Sampling farm	*Salmonella* spp.				*E. coli*			*C. perfringens*		
	Group	Mean	SD	*p*-value	Mean	SD	*p*-value	Mean	SD	*p*-value
Farm 1	Normal	0.89	1.19	0.2440	0.53	1.12	0.6704	0.39	0.64	*0.0001*
	Diarrhea	0.30	0.69		0.29	0.92		3.00	0.18	
Farm 2	Normal	1.82	1.67	0.6569	0.86	1.97	0.8038	3.29	1.09	*0.0014*
	Diarrhea	1.29	1.69		0.9	1.8		5.06	1.02	
Farm 3	Normal	0.93	1.25	0.8245	1.39	1.11	0.6710	1.57	0.91	*0.0005*
	Diarrhea	0.83	1.37		1.76	1.86		3.08	0.68	
Farm 4	Normal	1.50	1.34	0.1476	1.87	1.49	0.9377	1.59	0.6	*0.0007*
	Diarrhea	0.63	1.09		2.23	2.49		2.62	0.53	
*Total*	Normal	1.28	1.38	0.0821	1.16	1.50	0.7752	1.71	1.32	* < 0.0001*

Normal: isolates from clinically healthy corn snakes without diarrhea; Diarrhea: isolates from corn snakes that met the case definition for diarrhea.

Bacterial counts were log-transformed before statistical analysis. Two-group comparisons were performed using the Wilcoxon rank-sum test. Statistical significance was set at *p* < 0.05.

### Toxinotype distribution among *C. perfringens* isolates

Toxinotype A was the predominant type among all *C. perfringens* isolates, followed by toxinotype G, while toxinotypes B and D were each detected in a single isolate. The distribution of toxinotypes did not differ significantly between healthy and diarrheic snakes (Table 3).

**Table 3:** Distribution of toxinotype in 24 *C. perfringens* isolates.

Toxinotype	Distribution		*p*-value
	Normal (*n* = 12)	Diarrhea (*n* = 12)	
A	50% (6/12)	58.33% (7/12)	*0.6820*
B	8.33% (1/12)	0% (0/12)	*>0.9999*
D	0% (0/12)	8.33% (1/12)	*>0.9999*
G	41.67% (5/12)	33.33% (4/12)	*>0.9999*

Normal: isolates from clinically healthy corn snakes without diarrhea; Diarrhea: isolates from corn snakes that met the case definition for diarrhea; n: sample size.

Differences in detection rates between normal and diarrheic groups were analyzed using the Chi-square test. Fisher's exact test was applied when expected cell counts were insufficient. Statistical significance was set at *p* < 0.05.

### Comparison of virulence gene carriage rates and gene copy numbers between healthy and diarrheic isolates

**Figure 2:**
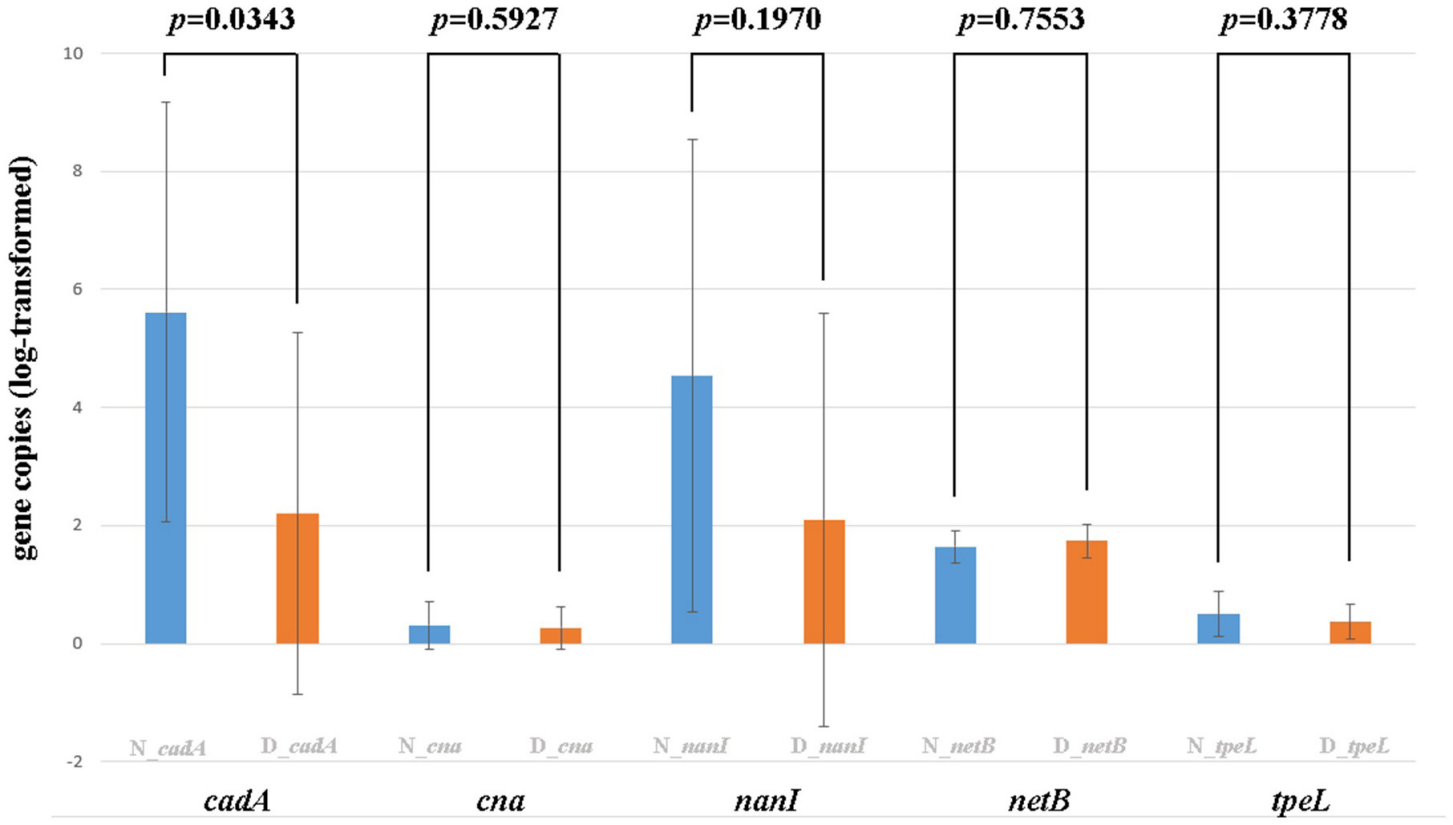
*Differential virulence gene copies between C. perfringens isolates from normal* ***(N)*** *and diarrhea* ***(D)*** *corn snakes*. Statistical analyses were performed using the Wilcoxon rank-sum test or two-sample *t* test, depending on data normality. Statistical significance was set at *p* < 0.05.

The carriage rates of virulence-associated genes (*cadA, cna, nanI, netB*, and *tpeL*) did not differ significantly between isolates from healthy and diarrheic snakes (Table 4). However, quantitative analysis showed that the *cadA* gene copy number was significantly lower in isolates from diarrheic snakes compared with those from healthy snakes (*p* = 0.0343). In contrast, no significant differences were observed in the copy numbers of *cna, nanI, netB*, or *tpeL* (all *p* > 0.05; Figure 2).

**Table 4:** Virulence gene carriage rate in 24 *C. perfringens* isolates.

Gene	Carriage rate		*p*-value
	Normal (*n* = 12)	Diarrhea (*n* = 12)	
*cadA*	91.67% (11/12)	66.67% (8/12)	*0.3168*
*cna*	50% (6/12)	41.67% (5/12)	*>0.9999*
*nanI*	58.33% (7/12)	33.33% (4/12)	*0.4136*
*netB*	100% (12/12)	100% (12/12)	*>0.9999*
*tpeL*	75% (9/12)	75% (9/12)	*>0.9999*

Normal: isolates from clinically healthy corn snakes without diarrhea; Diarrhea: isolates from corn snakes that met the case definition for diarrhea; n: sample size.

Differences in detection rates between normal and diarrheic groups were analyzed using the Chi-square test. Fisher's exact test was applied when expected cell counts were insufficient. Statistical significance was set at *p* < 0.05.

## Discussion

This study provides field-based evidence supporting an association between intestinal overgrowth of *C. perfringens* and fatal diarrhea in corn snakes across multiple breeding farms. Unlike *Salmonella* spp. and *E. coli*, which were detected in both healthy and diseased animals without significant differences, *C. perfringens* showed a significant association with disease status through both increased prevalence and elevated rectal bacterial loads. These findings suggest that pathogen burden, rather than simple presence, may contribute to disease expression in captive reptile systems. Similar associations between increased *C. perfringens* loads and enteric disease have been reported in sheep, lambs, and dogs (16–18), whereas findings in pigs and horses have been less consistent (19, 20). Such variability indicates the influence of host-specific factors in shaping the pathogenic potential of *C. perfringens* across different animal species.

*C. perfringens* is widely recognized as a commensal organism in the gastrointestinal tract of many animal species, and disease is often associated with quantitative expansion beyond baseline levels. Comparable load-dependent relationships have been described in poultry, calves, and sheep (21–23). In the present study, diarrheic snakes consistently exhibited higher rectal bacterial loads compared with healthy individuals across multiple facilities. This consistent pattern supports the observed association and suggests that quantitative assessment may provide more clinically relevant information than simple detection when evaluating diarrhea in captive corn snakes.

This study did not identify toxinotype F isolates carrying the *cpe* gene, which has been associated with diarrheal disease in reptiles (5) and humans (24, 25). The absence of *cpe*-positive isolates suggests that the strains identified in this study may differ from those typically implicated in enterotoxin-associated disease. However, their zoonotic potential cannot be ruled out. Toxinotyping did not differentiate isolates from healthy and diseased snakes, indicating that classical toxin classification alone may not explain disease expression in this host. This finding is consistent with reports from other animal systems, such as dogs and cattle (26, 27), where disease severity appears to depend on multifactorial interacting factors rather than toxinotype alone.

No significant differences in carriage rate or gene copy number were observed for *cna, nanI, netB*, or *tpeL* between isolates from healthy and diarrheic snakes. Although these genes have been implicated in enteric disease in other hosts, their distribution in the present study did not correspond with clinical status. Notably, isolates from diarrheic snakes exhibited numerically lower *nanI* detection rates and gene copy numbers, although these differences were not statistically significant. The *nanI* gene encodes a sialidase that degrades mucin and releases sialic acids, which can serve as nutrient sources for *C. perfringens* and support bacterial growth and colonization in the intestine (28, 29). The association between *nanI* activity and *C. perfringens* overgrowth has been demonstrated in experimental mouse models (9). However, the pattern observed in corn snakes suggest that intestinal expansion of *C. perfringens* in this host may not primarily rely on the *nanI*-mediated sialic acid pathway (30). Alternative mechanisms, including the potential involvement of other sialidases (31), or metabolic pathways, may contribute to bacterial persistence (32, 33). Further functional studies are required to clarify these mechanisms.

Among the virulence genes evaluated, *cadA* was the only gene demonstrating a statistically significant difference between groups, with lower gene copy numbers observed in isolates from diarrheic snakes. The *cadA* gene encodes an extracellular DNase involved in biofilm degradation and bacterial dispersal (11, 12). Reduced *cadA* abundance may be associated with altered biofilm dynamics, potentially influencing the balance between bacterial attachment and dispersal within the intestinal environment. Similar associations between reduced DNase activity, increased biofilm stability, and prolonged infection have been described in porcine and avian models of enteric disease (34–37).

In the present study, diarrheic snakes exhibited persistent diarrhea with minimal intestinal contents, yet rectal swabs consistently yielded high bacterial loads, often accompanied by epithelial material. These observations are compatible with sustained mucosal colonization. In contrast, isolates from healthy snakes showed higher *cadA* copy numbers, which may be associated with increased dispersal and shedding. However, gene copy numbers do not directly reflect gene expression or enzymatic activity. Further functional studies are required to determine whether altered *cadA*-mediated biofilm regulation contributes to disease persistence in corn snakes.

In conclusion, fatal diarrhea in captive corn snakes was associated with increased rectal colonization by *C. perfringens*, whereas toxinotype distribution did not differ between healthy and diseased snakes. Virulence gene profiling revealed patterns distinct from those described in other hosts, supporting host-specific variation in pathogenic mechanisms. Reduced *cadA* gene copy numbers were observed in isolates from diarrheic snakes, suggesting that altered colonization dynamics may contribute to disease expression. Overall, these findings provide field-based evidence supporting a potential role of *C. perfringens* burden in reptile enteric disease and establish a basis for future functional studies.

## Data Availability

The original contributions presented in the study are included in the article/Supplementary material, further inquiries can be directed to the corresponding author.
