# Supplementary material for: Intestinal overgrowth of Clostridium perfringens and reduced cadA gene copy number characterize fatal diarrhea in captive corn snakes (Pantherophis guttatus)
Source: Front Vet Sci. 2026 May 25;13:1812888. doi: 10.3389/fvets.2026.1812888 (PMC13243109; doi:10.3389/fvets.2026.1812888)
Supplement: Supplementary file 1 [file Table_1.docx]

***Supplementary data***

**Table S1. Primers used for *C. perfringens* toxinotyping and virulence gene profiling**

| **Gene** | **GenBank**  **accession No.** | **Forward primer (5'→3')** | **Reverse primer (5'→3')** | **Product size**  **(bp)** | **Reference** |
| --- | --- | --- | --- | --- | --- |
| *16S rRNA* | Y12669 | CATCATTCAACCAAAGGAGCAATCC | CATTATCTTCCCCAAAGACAGAGC | 262 | (Yang, et al., 2018) |
| *cpa* | KP143661 | CTGACACAGGGGAATCACAAATAAG | CTCTCAACGGCAGTTACATTAGCAG | 163 | *This study* |
| *cpb* | L13198 | ACAGACAGATCATTCAACCTCTAAAGC | AGATTCTTCAGTACCATTAGGAGCAG | 247 | *This study* |
| *etx* | JX010451 | AACTGCAACTACTACTCATACTGTGG | TGGTACTAGTATATCTTGTGAAGGGAC | 178 | *This study* |
| *iap* | X73562 | CGCTGGTGACCCATATACAGATTATC | ATCCAGCACTAATAGATACTCCAACTG | 236 | *This study* |
| *cpe* | - | ATAGATAAAGGAGATGGTTGGA | CCATATTCTACAGATGCTTGTA | 178 | (Mahamat Abdelrahim, et al., 2019) |
| *cna* | - | GGTGGATGGGCAACATTTAC | CCTTGCTTGGATTCACCAGT | 183 | (Lepp, et al., 2021) |
| *cadA* | - | AGATCGAGCCCATAGAAGCTG | ACATCTCCCAAATACAGCTTCC | 198 | (Mahamat Abdelrahim, et al., 2019) |
| *nanI* | - | AAGGTAAACAATCTAGTGCTGT | TCTATTATCATTTGGAGCTTCTC | 82 | (Mahamat Abdelrahim, et al., 2019) |
| *netB* | GU433338 | TGATACCGCTTCACATAAAGGGTTGG | ATTAGTTTCAGGCCATTTCATTTTTCCG | 169 | (Yang, et al., 2018) |
| *tpeL* | - | GTGCCAATTGCAGGTATATCAAG | ATCCTCCTTCCATTGCCCATA | 247 | (Mahamat Abdelrahim, et al., 2019) |

Abbreviations: 16S rRNA, 16S ribosomal RNA gene; *cpa*, alpha toxin gene; *cpb*, beta toxin gene; *etx*, epsilon toxin gene; *iap*, iota toxin component A gene; *cpe*, enterotoxin gene; *cna*, collagen adhesion gene; *cadA*, adhesion-associated gene; *nanI*, neuraminidase gene; *netB*, necrotic enteritis B-like toxin gene; *tpeL*, toxin perfringens large gene.

“-” indicates that no GenBank accession number was available in the original reference.

Gene annotations were based on published literature and GenBank records.

**
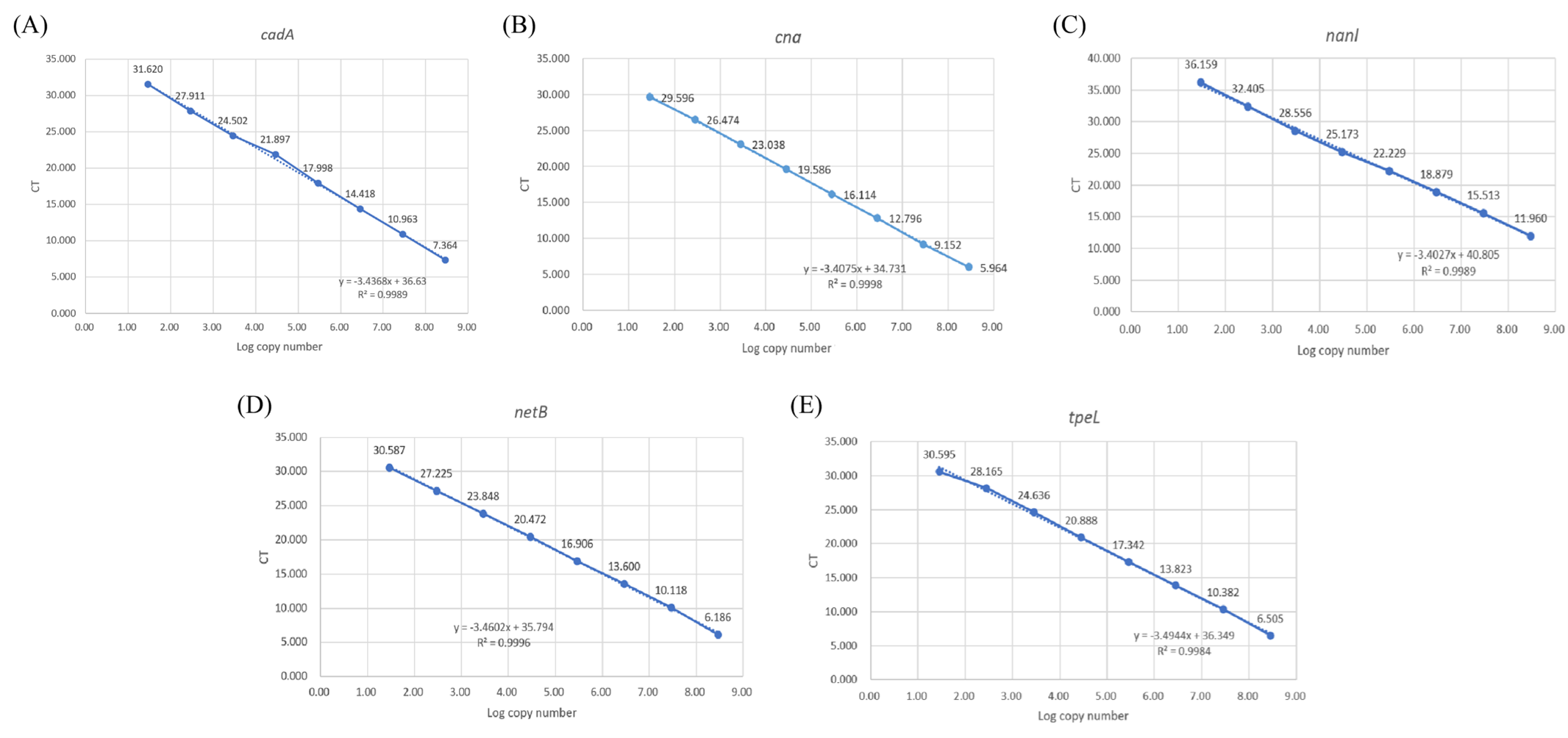
**

**Figure S1. Standard curve of virulence gene copies by quantitative real-time polymerase chain reaction.** (A) *cadA* gene: R2: 0.9989; amplification efficiency (AE): 0.9542; (B) *cna* gene: R2: 0.9998; AE: 0.9655 ; (C) *nanI* gene: R2: 0.9989; AE: 0.9674; (D) *netB* gene: R2: 0.9995; AE: 0.95; (E) *tpeL* gene: R2: 0.9984; AE: 0.9327.

**References**

1. Lepp, D., Y. Zhou, S. Ojha, I. Mehdizadeh Gohari, J. Carere, C. Yang, J. F. Prescott, and J. Gong. 2021. *Clostridium perfringens* produces an adhesive pilus required for the pathogenesis of necrotic enteritis in poultry. J Bacteriol 203. doi 10.1128/jb.00578-20
2. Mahamat Abdelrahim, A., N. Radomski, S. Delannoy, S. Djellal, M. Le Négrate, K. Hadjab, P. Fach, J. A. Hennekinne, M. Y. Mistou, and O. Firmesse. 2019. Large-scale genomic analyses and toxinotyping of *Clostridium perfringens* implicated in foodborne outbreaks in France. Front. Microbiol. 10:777. doi 10.3389/fmicb.2019.00777
3. Yang, W. Y., C. H. Chou, and C. Wang. 2018. Characterization of toxin genes and quantitative analysis of *netB* in necrotic enteritis (NE)-producing and non-NE-producing *Clostridium perfringens* isolated from chickens. Anaerobe 54:115-120. doi 10.1016/j.anaerobe.2018.08.010
